# Neutrophil-derived apoptotic bodies function as decoy signals to preserve bone integrity during infectious osteomyelitis

**DOI:** 10.1038/s41413-026-00571-z

**Published:** 2026-08-25

**Authors:** Qixiu Yu, Ying Qu, Jie Zhang, Ziyang Zhang, Shuquan Guo, Fei Luo, Ce Dou

**Affiliations:** 1 Department of Orthopedics, Southwest Hospital, Army Medical University, Chongqing, China; 2 Department of Orthopaedics, The First Affiliated Hospital of Chongqing Medical University, Chongqing, China

**Keywords:** Bone, Pathogenesis

## Abstract

Bone infection represents a pathological intersection of immunity and skeletal remodeling, where inflammatory responses drive excessive bone loss. Neutrophils dominate the early immune infiltration during infection, yet how neutrophil fate within infected bone marrow influences disease progression remains poorly understood. Here, we identify neutrophil-derived apoptotic bodies (Neut-ABs) as key mediators of bone protection during infectious osteomyelitis. Bone infection triggers extensive neutrophil apoptosis, resulting in the accumulation of Neut-ABs. Integrated transcriptomic and proteomic profiling of these Neut-ABs revealed significant enrichment of TLR2 and the sialyltransferase ST3GAL1, and lectin blotting confirmed that TLR2 is modified with α2,3-linked sialic acids. Mechanistically, α2,3-sialylated TLR2 on Neut-ABs functions as a molecular decoy that competitively interferes with Siglec15–TLR2 interactions in osteoclast precursors, thereby preventing precursor fusion and suppressing bone-resorptive activity. This inhibitory effect requires both the formation and sialylation of Neut-ABs, as it was abolished in Fas-deficient (MRL/Lpr) mice, upon neuraminidase-mediated desialylation, or when using Neut-ABs derived from *Tlr2*^⁻/⁻^ mice. Therapeutic delivery of Neut-ABs markedly attenuates osteolysis in infected mice without altering bacterial clearance. These findings uncover a neutrophil-to-bone signaling axis in which sialylated vesicular decoys selectively suppress osteoclast activation, illuminating a new paradigm linking innate immune turnover to skeletal homeostasis.

## Introduction

Bone infection represents a unique pathological interface between the immune and skeletal systems, where host defense and tissue remodeling occur in the same confined microenvironment.^1–5^ During infection, inflammatory cytokines and recruited immune cells disrupt the balance of bone formation and resorption, leading to excessive osteoclast activation and progressive bone destruction.^6–9^ Among these immune cells, neutrophils dominate the early infiltrate and are essential for bacterial clearance through phagocytosis, degranulation, and NET formation.^10,11^ However, their massive turnover within infected bone marrow also generates abundant cellular remnants and metabolic stress, the consequences of which on skeletal integrity remain poorly understood.^12–14^

Neutrophils recruited to sites of bone infection experience intense activation followed by rapid apoptosis, a process essential for resolving inflammation and limiting collateral tissue damage.^15^ Within the confined marrow environment, this accelerated turnover releases large numbers of membrane-enclosed vesicles known as apoptotic bodies (ABs), which contain diverse proteins, lipids, and glycosylated surface receptors. Once regarded as inert remnants of dying cells, ABs are now recognized as active mediators of intercellular communication that modulate immune and reparative responses across various tissues.^16,17^ Despite this emerging paradigm,^18,19^ it remains unknown whether neutrophil apoptosis during bone infection gives rise to such vesicles within the marrow niche, and if so, whether these neutrophil-derived apoptotic bodies (Neut-ABs) contribute to infection-associated pathological bone loss. Addressing this question is critical to understanding how innate immune turnover shapes skeletal integrity under infectious stress.

Bacterial infection profoundly reprograms neutrophil surface glycosylation, altering the repertoire and linkage of sialylated glycans that decorate key immune receptors.^20,21^ Such infection-driven remodeling enhances α2,3-linked sialylation on membrane proteins involved in adhesion, recognition, and signaling, thereby modulating neutrophil activation and lifespan within inflamed tissues.^22^ These sialic acid modifications also serve as molecular cues for Siglec family receptors on myeloid and stromal cells, functioning as a regulatory language that shapes cellular communication and inflammatory resolution.^23–25^ During apoptosis, the composition of these surface glycans is further reorganized, and the resulting apoptotic bodies inherit the sialylated membrane components of their parental cells. This raises the intriguing possibility that Neut-ABs preserve infection-induced sialylation patterns capable of engaging Siglec receptors in the bone microenvironment. Whether such glycan-dependent recognition governs the crosstalk between Neut-ABs and surrounding skeletal cells during infection remains unknown.

Here, we identify and characterize Neut-ABs as key mediators linking infection-induced immune turnover to skeletal preservation. Using integrated transcriptomic, proteomic, and biochemical analyses, we show that Neut-ABs are enriched in membrane proteins bearing α2,3-sialylated TLR2, which functions as a molecular decoy to disrupt Siglec15–TLR2 signaling in osteoclast precursors. Functional assays in vitro and in mouse models of infectious osteomyelitis demonstrate that Neut-ABs suppress osteoclast fusion and bone resorption, thereby protecting against infection-driven bone loss without compromising bacterial clearance. These findings identify a neutrophil-to-bone apoptotic axis that protects against infection-induced bone loss.

## Results

### Formation and characterization of neutrophil-derived apoptotic bodies

**Fig. 1:**
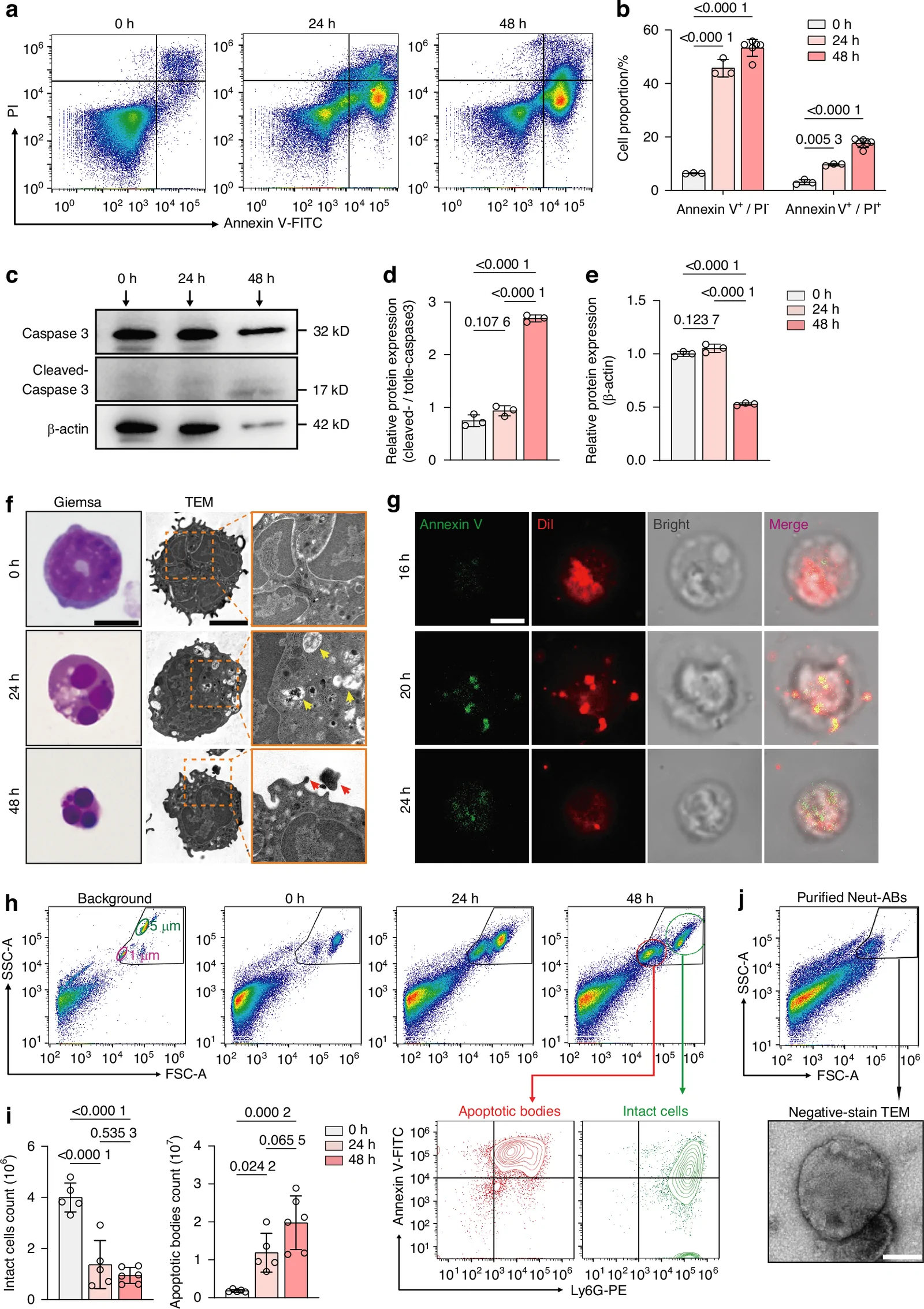
Neutrophil apoptosis and generation of apoptotic bodies. Flow cytometry of bone marrow neutrophils cultured in vitro (0–48 h), showing representative Annexin V/PI plots (**a**) and quantification of early (Annexin V⁺/PI⁻) and late apoptotic (Annexin V⁺/PI⁺) neutrophils over time (**b**, *n* = 3–6). **c**–**e** Western blot analysis of neutrophils at different culture time points, showing caspase-3 cleavage and β-actin degradation (30 µg protein per lane, *n* = 3). **f** Representative Wright-Giemsa staining and TEM images of neutrophils. Yellow arrows indicate cytoplasmic vacuoles, and red arrows point to budding apoptotic bodies. Scale bar = 5 µm. **g** Representative time-lapse images of neutrophils cultured in vitro, showing membrane dynamics. Annexin V is shown in green, and DiI membrane dye is shown in red. Scale bar = 5 µm. **h**–**i** Flow cytometry analysis of intact neutrophils and apoptotic bodies in the in vitro cultured neutrophil system. Representative plots (**h**); the background control contains culture medium with 1 μm and 5 μm beads to indicate particle size. Quantification of intact cells and apoptotic bodies over time (**i**, *n* = 5–6). **j** Representative flow cytometry plots of purified Neut-ABs, along with negative-stain TEM images showing their morphology. Scale bar = 200 nm. Data are presented as mean ± SD, with statistical significance (exact *P* values shown in the figure) determined by one-way ANOVA followed by Tukey’s multiple comparisons test (**b**, **d**, **e** and **i**). See also Fig. S1

To comprehensively characterize neutrophil apoptosis, we first isolated and purified neutrophils from mouse bone marrow using a negative magnetic selection method (Fig. S1a), which preserves cell integrity and minimizes activation. During in vitro culture (0–48 h), these cells exhibited a strong propensity for spontaneous apoptosis, consistent with previous reports,^26^ as evidenced by a progressive increase in Annexin V⁺/PI⁺ events detected by flow cytometry (Fig. 1a, b). Western blot analysis using 30 µg protein per lane revealed caspase-3 cleavage, together with degradation of β-actin, corroborating apoptotic activation (Fig. 1c–e).

We next examined morphological changes at three time points using Wright-Giemsa staining and transmission electron microscopy (TEM) (Fig. 1f). Freshly isolated bone marrow neutrophils (0 h) displayed characteristic nuclear morphologies, including ring-shaped nuclei and segmented forms (Fig. S1b), the latter indicative of advanced maturation. By 24 h, apoptotic neutrophils exhibited cytoplasmic vacuolization, loss of surface microvilli, nuclear pyknosis and fragmentation into several rounded bodies (Fig. 1f). As apoptosis progressed (24–48 h), membrane blebs became increasingly prominent (Fig. 1f) and subsequently detached as apoptotic bodies of heterogeneous size (Fig. S1c).

Time-lapse imaging captured the formation of these membrane blebs, which were visualized with the membrane dye DiI, and their apoptotic nature was confirmed by Annexin V staining (Fig. 1g). Additionally, flow cytometric analysis identified vesicular particles positive for both Annexin V and Ly6G, confirming their identity as neutrophil-derived apoptotic bodies (Neut-ABs; Fig. 1h). Quantitative analysis further revealed a time-dependent decrease in intact neutrophils, accompanied by a reciprocal increase in apoptotic bodies, highlighting the progressive nature of apoptosis in this population (Fig. 1i). Finally, Neut-ABs were purified using standard differential centrifugation^27^ (Fig. S1d) and subsequently visualized by negative-stain TEM and confocal microscopy (Fig. 1j; Fig. S1e), confirming their characteristic morphology and size distribution (Fig. S1f, S1g).

### Neut-AB deficiency aggravates infection-induced bone destruction

Having completed the characterization of Neut-ABs in vitro, we next evaluated their functional relevance in infectious osteomyelitis. We established the osteomyelitis model based on a previously reported protocol^28^ with minor modifications (Fig. S2a; see Methods). Infection led to a pronounced increase in neutrophil apoptosis (Fig. S2b).

**Fig. 2:**
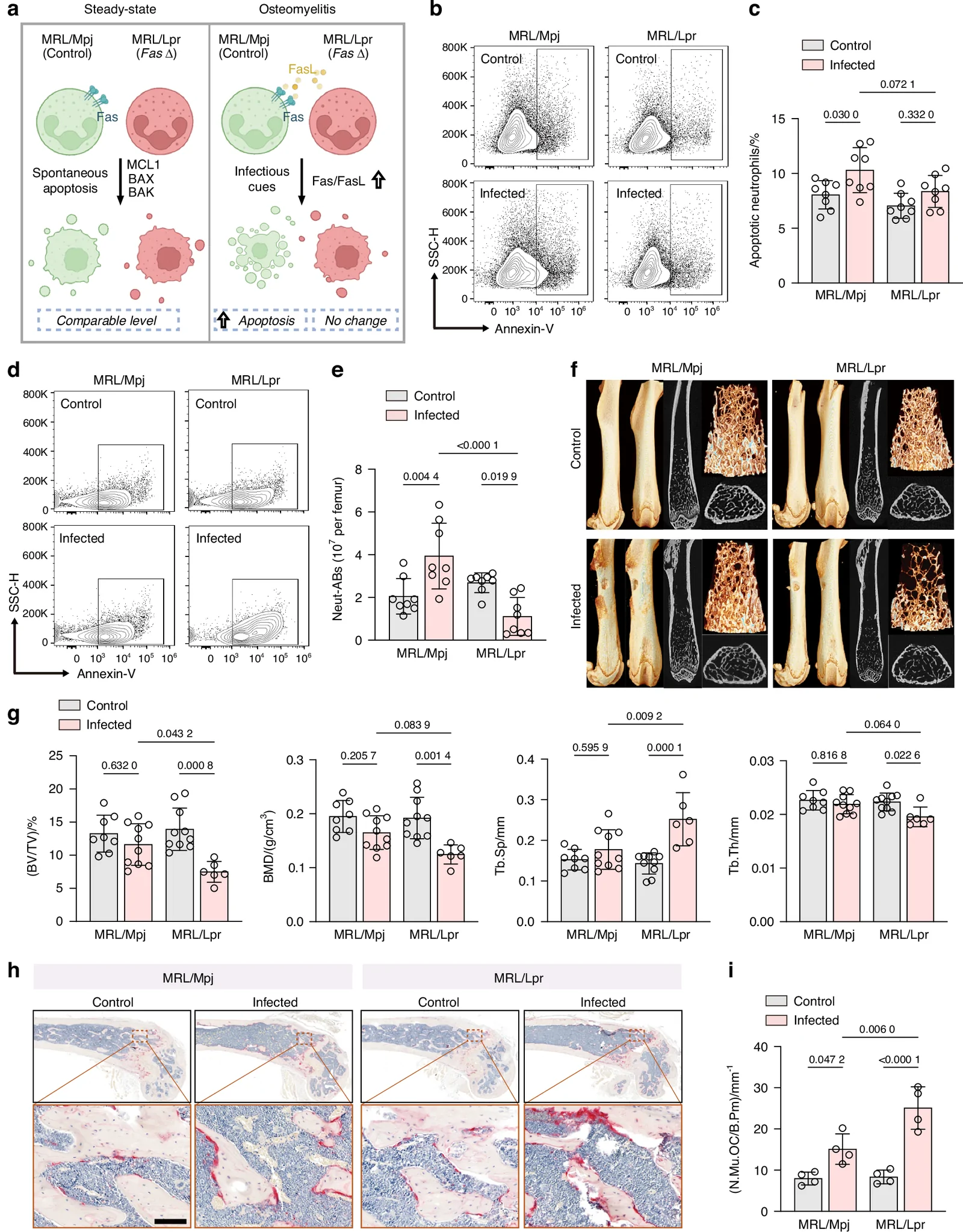
Neut-ABs deficiency exacerbates osteomyelitis-induced bone loss in MRL/Lpr mice in vivo. **a** Schematic illustrating differences in apoptosis propensity of bone marrow neutrophils from MRL/Mpj (green) and MRL/Lpr (red) mice under steady-state and *S. aureus*–induced osteomyelitis. **b**–**e** Flow cytometry analysis of bone marrow neutrophils from MRL/Mpj and MRL/Lpr mice under steady-state and *S. aureus*–induced osteomyelitis conditions at 1 day post-infection; gating strategy is provided in Fig. S2E. Representative plots showing apoptotic neutrophils (**b**). Quantification of apoptotic neutrophils (**c**, *n* = 8). Representative plots showing neutrophil-derived apoptotic bodies (d). Quantification of Neut-ABs per femur (**e**, *n* = 8). **f**, **g** Representative micro-CT images of femurs 15 days after S. aureus infection (**f**). Quantitative micro-CT analysis of bone parameters, including BV/TV, BMD, Tb.Sp, and Tb.Th (**g**, *n* = 6–10). **h**–**i** Representative TRAP-stained histological sections showing osteoclasts (**h**). Quantification of osteoclast numbers (**i**, *n* = 4), expressed as N.Mu.OC/B.Pm (number of multinucleated osteoclasts per bone perimeter). Scale bar = 100 µm. Data are presented as mean ± SD, with statistical significance (exact *P* values shown in the figure) determined by one-way ANOVA followed by Tukey’s multiple comparisons test (**c**, **e**, **g** and **i**). See also Figs. S2 and S3

To investigate the function of Neut-ABs in vivo, *Staphylococcus aureus*-induced osteomyelitis was established in MRL/Lpr mice (hereafter Lpr), which carry a Fas mutation leading to defective apoptosis, together with MRL/Mpj mice (hereafter Mpj) as background-matched controls (Fig. S2c–S2f). We also validated the robustness of our flow cytometric gating strategy when extending its application from in vitro to in vivo analyses (Fig. S2g and Fig. 1h). Although Lpr mice carry a *Fas* deficiency, neutrophils are short-lived cells that primarily undergo spontaneous apoptosis via intrinsic pathways under steady-state conditions, with minimal contribution from Fas signaling.^29,30^ Differences between Lpr and Mpj neutrophils become apparent only under infection-induced stress, when FasL expression is upregulated locally^30–34^ (Fig. 2a), making Lpr mice particularly suitable for studying infection-dependent Neut-ABs deficiency.

Following *Staphylococcus aureus* infection, both Lpr and Mpj mice showed a reduction in bone marrow neutrophils (Fig. S3a, S3b). However, neutrophil apoptosis was robustly upregulated in Mpj mice, whereas Lpr neutrophils failed to exhibit this increase (Fig. 2b, c), indicating an infection-induced, Fas-dependent apoptotic defect in Lpr neutrophils. Correspondingly, Neut-ABs accumulated substantially in the bone marrow of infected Mpj mice but were largely absent in Lpr mice (Fig. 2d, e). Bacterial burden in Mpj mice was slightly higher than in Lpr mice, although the difference did not reach statistical significance (Fig. S3c). Serum TNF-α and IL-1β levels were comparable between Lpr and Mpj mice throughout the experimental period, and renal histology showed no overt pathology (Fig. S3d, S3e). Micro-CT analysis revealed pronounced bone loss in Lpr mice following *Staphylococcus aureus* infection, reflected by decreased bone mineral density (BMD), reduced bone volume fraction (BV/TV), diminished trabecular thickness (Tb.Th), and increased trabecular separation (Tb.Sp) (Fig. 2f, g), occurring in parallel with the marked reduction of Neut-ABs. Histological analysis with TRAP (tartrate-resistant acid phosphatase) staining revealed that the enhanced bone destruction in Lpr mice was associated with increased osteoclast numbers (Fig. 2h, i), rather than alterations in osteoblast activity, as reflected by immunohistochemical (IHC) analysis of osteocalcin (OCN; a marker of osteoblast activity) (Fig. S3f, S3g). Together, these findings suggest that the infection-induced deficiency of Neut-ABs in Lpr mice contributes to enhanced osteoclast-mediated bone destruction, highlighting the potential role of neutrophil apoptosis and Neut-ABs generation in the maintenance of bone integrity during infectious osteomyelitis.

### Integrated omics reveal membrane protein enrichment underlying the communication potential of Neut-ABs

**Fig. 3:**
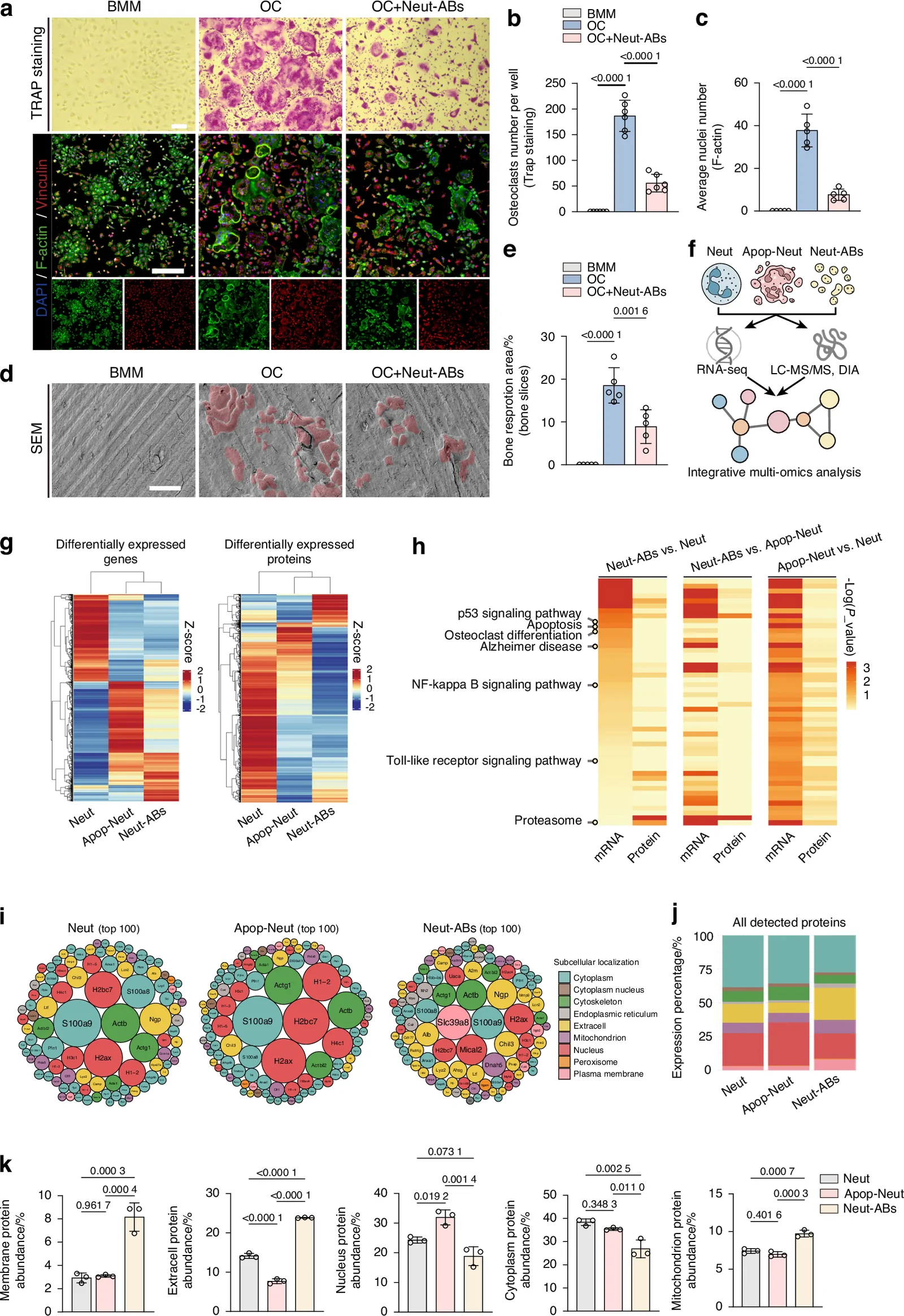
Membrane components of neutrophil-derived apoptotic bodies mediate suppression of osteoclastogenesis. **a**–**c** In vitro osteoclastogenesis assay. Representative TRAP-stained and immunofluorescence images of bone marrow–derived macrophages (BMM) and osteoclasts (OC), with DAPI in blue, phalloidin-labeled F-actin in green, and Vinculin in red (**a**, scale bar = 200 µm). Quantification of osteoclast numbers per well (**b**, *n* = 6) and average number of nuclei per osteoclast (**c**, *n* = 5). (**d–e**) Representative scanning electron microscopy (SEM) images of bone slices after resorption assay, with resorption pits highlighted in red pseudocolor (**d**, scale bar = 50 µm). Quantification of resorption area (**e**, *n* = 5). **f** Schematic of experimental workflow for integrated transcriptomic and proteomic profiling of neutrophils (Neut), apoptotic neutrophils (Apop-Neut), and neutrophil-derived apoptotic bodies (Neut-ABs). **g** Heatmap showing hierarchical clustering of differentially expressed genes and proteins across the three groups. Expression levels were normalized by Z-score for visualization. **h** Joint KEGG pathway enrichment analysis integrating transcriptomic and proteomic data, showing the top 50 enriched pathways. **i** Top 100 most abundant proteins in each sample group, colored by subcellular localization and sized according to relative abundance. **j**–**k** Subcellular localization analysis of proteins. Bar graph showing the distribution of all detected proteins across each sample group (**j**). Quantitative comparison of protein abundance among five subcellular compartments (**k**). Data are presented as mean ± SD, with statistical significance (exact *P* values shown in the figure) determined by one-way ANOVA followed by Tukey’s multiple comparisons test (**b**, **c**, **e** and **k**). See also Fig. S4

Building on the in vivo findings that increased osteoclast activity underlies the exacerbated bone destruction in Neut-ABs-deficient mice, we next assessed whether Neut-ABs directly modulate osteoclastogenesis. Co-culture of osteoclast precursors with Neut-ABs during in vitro osteoclast formation demonstrated that while TRAP positivity was maintained, the formation of multinucleated osteoclasts, the size of F-actin rings and bone resorption activity were all significantly reduced (Fig. 3a–e), indicating that Neut-ABs directly inhibit osteoclast differentiation and attenuate their resorptive function.

To delineate the molecular signatures underlying Neut-ABs identity and function, we performed integrated transcriptomic and proteomic profiling of neutrophils (Neut), apoptotic neutrophils (Apop-Neut), and neutrophil-derived apoptotic bodies (Neut-ABs) (Fig. 3f). Principal component analysis (PCA) revealed distinct grouping of the three sample types with consistent within-group similarity (Fig. S4a). Hierarchical clustering of differentially expressed genes (DEGs) and proteins (DEPs) showed that Neut-ABs share expression patterns more closely with apoptotic neutrophils than with fresh neutrophils (Fig. 3g), consistent with our previous study demonstrating that apoptotic bodies retain molecular features similar to their parent cells, supporting the apoptotic origin of Neut-ABs. Transcriptomic analysis revealed canonical apoptotic signatures, particularly prominent in the neutrophil lineage: downregulation of the anti-apoptotic gene *Mcl1*,^35,36^ upregulation of the pro-apoptotic gene *Bax* and *Fas*/*Fasl* signaling,^36^ and enrichment of senescence-associated modules. In contrast, necroptosis mediators (*Ripk1*/*Ripk3*/*Mlkl*)^37^ were not induced, distinguishing neutrophil apoptosis from necroptosis (Fig. S4b). Other reported redundant regulators^36^ showed no consistent changes (Fig. S4c). Proteomic profiling corroborated these apoptotic and senescent signatures (Fig. S4d). Joint KEGG enrichment of DEGs and DEPs highlighted p53 signaling and apoptosis, as well as pathways associated with degenerative disorders, including Alzheimer’s disease, which may reflect shared molecular signatures previously observed in neuronal disorders.^38,39^ Notably, pathways related to osteoclast differentiation were also enriched, aligning with our in vivo observations (Fig. 3h). Transcriptome–proteome correlation progressively decreased from neutrophils to apoptotic neutrophils and was lowest in Neut-ABs (Fig. S4e; ρ = 0.311 → 0.287 → 0.262). This indicates a progressive uncoupling of transcript and protein profiles, suggesting that functional insights into Neut-ABs are more reliably derived from protein-level analyses.

We then focused on the proteomic dataset to explore molecular features underlying Neut-ABs function. During neutrophil apoptosis and Neut-ABs formation, most proteins were generally downregulated (Fig. S4f, S4h). However, visualization of the top 100 most abundant proteins revealed a pronounced enrichment of membrane and extracellular proteins (Fig. 3i; Fig. S4g), a trend that persisted when extended to the entire proteome (Fig. 3j, k; Fig. S4i). Given the pivotal role of membrane proteins in intercellular communication,^40–42^ this enrichment likely underlies the ability of Neut-ABs to interact with surrounding cells and mediate their protective functions.

### Sialylated TLR2 enables osteoclast recognition of Neut-ABs

Osteoclasts are terminally differentiated cells of the monocyte–macrophage lineage. During differentiation, precursor fusion generates multinucleated cells with well-organized F-actin rings, which are essential for forming localized acidic resorption lacunae and enabling bone resorption.^43^ Post-translational glycosylation, particularly sialylation, serves as a critical mediator of intercellular communication and self/non-self recognition.^44^ Previous studies, including ours, have demonstrated that sialylation on osteoclast precursors is essential for precursor–precursor fusion (Fig. S5a), with the Siglec15–Tlr2 axis acting as a central regulator.^45–48^

**Fig. 4:**
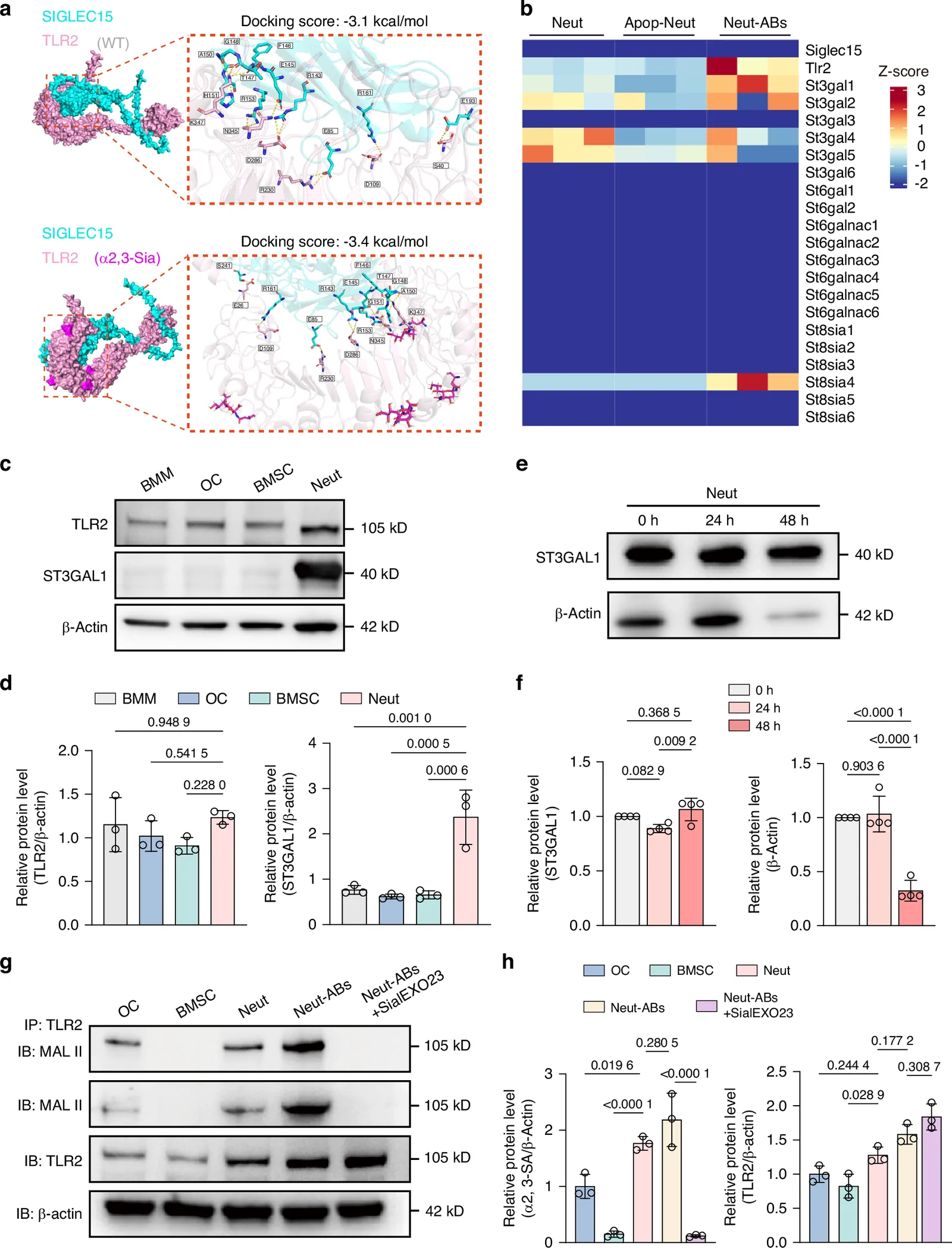
Molecular features of neutrophil apoptotic bodies related to sialylation and Tlr2 expression. **a** molecular docking illustrating the predicted interaction of Siglec15 with WT or α2,3-sialylated TLR2. Siglec15 is in cyan-green, TLR2 in soft pink, and α2,3-linked sialic acids in purple. **b** Heatmap showing the expression of Siglec15, Tlr2, and sialyltransferase family proteins across three sample groups, with expression levels normalized by Z-score for visualization. **c**, **d** Western blot analysis of TLR2 and ST3GAL1 protein expression in different primary cells, with β-actin as a loading control (*n* = 3). **e**, **f** Western blot analysis of neutrophils at different culture time points, showing ST3GAL1 and β-actin (30 µg protein per lane, *n* = 4). **g**–**h** Representative Western blot, lectin blot, and immunoprecipitation images of protein expression and glycosylation in lysates from different experimental groups (**g**) with MAL II used to detect α2,3-linked sialic acids, and corresponding quantification of band intensity (**h**, *n* = 3). Data are presented as mean ± SD, with statistical significance (exact *P* values shown in the figure) determined by one-way ANOVA followed by Tukey’s multiple comparisons test (**d**, **f** and **h**). See also Fig. S5

During osteoclast differentiation, the sialic acid–binding receptor Siglec15 is progressively upregulated. Tlr2 has been reported as a candidate Siglec15–binding partner based on proximity-labeling experiments^49^ (Fig. S5b). Their interaction requires α2,3-linked sialylation mediated by *St3gal1*, rather than α2,6 or other linkages.^46^ Molecular docking indicated that wild-type Tlr2 and Siglec15 form 14 stable hydrogen bonds with a binding energy of −3.1 kcal/mol. Sialylation of Tlr2 induced local conformational rearrangements, enhancing the binding affinity to -3.4 kcal/mol (Fig. 4a). Genetic disruption of either Siglec15 or Tlr2 resulted in impaired osteoclast formation and osteopetrosis, underscoring the biological importance of this recognition pathway^46,50,51^ (Fig. S5c).

Given that neutrophils heavily rely on glycosylated surface proteins for migration, adhesion, and recognition,^20,52^ we next interrogated our multi-omics data for sialylation-related features. Transcriptomic heatmaps indicated that although neutrophils lack the osteoclast-specific molecule *Siglec15*, they express *Tlr2* along with multiple sialyltransferases (Fig. S5d). Proteomic profiling, which generally detects fewer proteins than transcriptomics, revealed robust expression of St3gal1, St3gal2, St3gal4, St3gal5, and St8sia4, with St3gal1 and Tlr2 enriched in Neut-ABs (Fig. 4b). Western blotting across bone-related cell types confirmed that Tlr2 is present in bone marrow macrophages (BMM), osteoclasts (OC), mesenchymal stem cells (BMSC), and neutrophils, with neutrophils showing the highest expression of St3gal1 (Fig. 4c, d). Despite progressive degradation of common housekeeping proteins such as β-actin during neutrophil apoptosis, ST3GAL1 protein remained largely stable, indicating preferential preservation throughout the apoptotic process (Fig. 4e, f).

We further validated sialylation at the protein level using Maackia amurensis lectin II (MAL II), which specifically binds to α2,3-linked sialic acid residues. Lectin blotting revealed an ~105 kD α2,3-sialylated band corresponding to glycosylated Tlr2 in osteoclasts, which was confirmed by immunoprecipitation (Fig. 4g, h). BMSCs expressed Tlr2 but lacked detectable α2,3-sialylation. In contrast, neutrophils exhibited abundant α2,3-sialylated Tlr2, which was further enriched in Neut-ABs. This modification could be removed by treatment with SialEXO 2-3 (a neuraminidase specific for α2,3-linked sialic acids) (Fig. 4g, h). Together with the proteomic enrichment of membrane proteins (Fig. 3i–k), these results indicate that neutrophils are highly sialylated and that Neut-ABs are enriched in α2,3-sialylated Tlr2, a modification likely facilitating selective recognition and interaction with osteoclast precursors.

### Sialylated TLR2 endows Neut-ABs with decoy activity against osteoclastogenesis

**Fig. 5:**
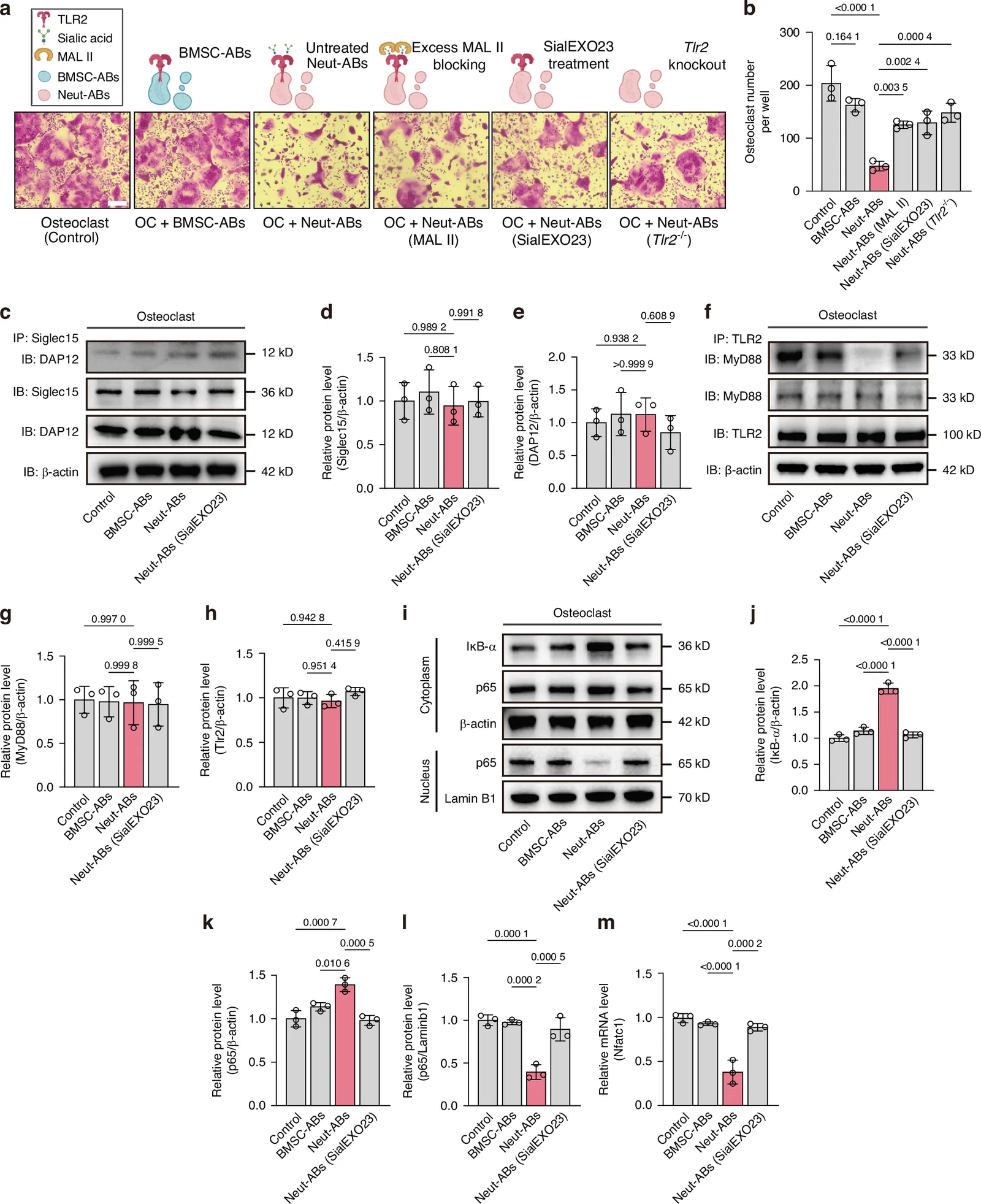
Neutrophil-derived apoptotic bodies interfere with TLR2–siglec15 interaction to suppress osteoclastogenesis. **a**, **b** *Schematic and representative TRAP-stained images of osteoclast precursors treated with apoptotic bodies from different sources or treatments* (a, scale bar = 200 µm), with corresponding quantification per well (**b**, *n* = 3). Representative IP and WB images of Siglec15 and DAP12 (**c**) with corresponding quantification (**d**, **e**, *n* = 3). β-actin and total protein were used as loading controls. Representative IP and WB images of TLR2 and MyD88 (**f**) with corresponding quantification (**g**, **h**, *n* = 3). β-actin and total protein were used as loading controls. **i**–**l** Western blot analysis of p65 and IκB in nuclear and cytoplasmic fractions of osteoclasts under different treatments (**i**). β-actin (cytoplasmic fraction) and Lamin B1 (nuclear fraction) were used as loading controls, with corresponding quantification (**j**–**l**, *n* = 3). **m** qPCR analysis of *Nfatc1* expression in osteoclasts under different treatments, with *Gapdh* as the internal control (*n* = 3). Data are presented as mean ± SD, with statistical significance (exact *P* values shown in the figure) determined by one-way ANOVA followed by Tukey’s multiple comparisons test (**b**, **d**, **e**, **g**, **h**, **i**, **j**, **k**, **l** and **m**). See also Fig. S6

We further explored the functional relevance of α2,3-sialylated Tlr2 on Neut-ABs by examining their impact on osteoclast precursor fusion. Previous lectin blot results indicated that BMSC lack detectable α2,3-sialylated Tlr2 (Fig. 4g, h). Accordingly, we used BMSC-derived apoptotic bodies (BMSC-ABs) as a negative control. Osteoclast precursors were exposed to untreated Neut-ABs, Neut-ABs pre-incubated with excess MAL II to block α2,3-sialic acids, Neut-ABs treated with SialEXO 2-3 to enzymatically remove sialic acids, or Neut-ABs derived from *Tlr2*^*-/-*^ mice. Consistent with the functional involvement of α2,3-sialylated Tlr2, BMSC-ABs had no effect on osteoclast fusion, whereas Neut-ABs strongly suppressed multinucleated cell formation. Importantly, blockade or removal of sialic acids, as well as Tlr2 deficiency, abrogated this inhibitory effect (Fig. 5a, b), indicating that α2,3-sialylation on Tlr2 is required for the decoy function of Neut-ABs.

Next, we investigated the signaling mechanisms underlying this inhibition by examining key molecular interactions in osteoclast precursors. Immunoprecipitation (IP) and western blot analyses revealed that Neut-ABs did not alter the abundance or binding of Siglec15 to its core adaptor protein DAP12 (Fig. 5c–e), nor did they affect downstream phosphorylation of Syk, a critical ITAM-motif-mediated signal (Fig. S6a–S6c). In contrast, while Neut-ABs had no effect on TLR2 or MyD88 protein expression, they significantly impaired the recruitment of MyD88 to TLR2 (Fig. 5f–h), which in turn prevented IκB-α degradation and limited nuclear translocation of p65, as confirmed by nuclear/cytoplasmic fractionation (Fig. 5i–l). PCR analysis further showed that Neut-ABs reduced Nfatc1 mRNA levels in osteoclasts (Fig. 5m), consistent with impaired NF-κB signaling and consequent downregulation of *Nfatc1*, the master regulator of osteoclast differentiation and function.^53^

Taken together, these findings demonstrate that α2,3-sialylated Tlr2 on Neut-ABs functions as a critical mediator of the decoy activity (Fig. S6d). During infection, Neut-ABs accumulate in the bone marrow and engage Siglec15 on osteoclast precursors in trans, thereby occupying the receptor and preventing the formation of functional Siglec15–TLR2 signaling complexes. Consequently, TLR2–MyD88 signaling is impaired, leading to suppression of downstream osteoclastogenic transcriptional programs. This mechanism provides a molecular basis for the protective effect of Neut-ABs on bone tissue during infectious osteomyelitis, highlighting a novel role for sialylated membrane proteins in modulating intercellular communication and osteoclastogenesis.

### Therapeutic delivery of Neut-ABs preserves bone integrity during infection

**Fig. 6:**
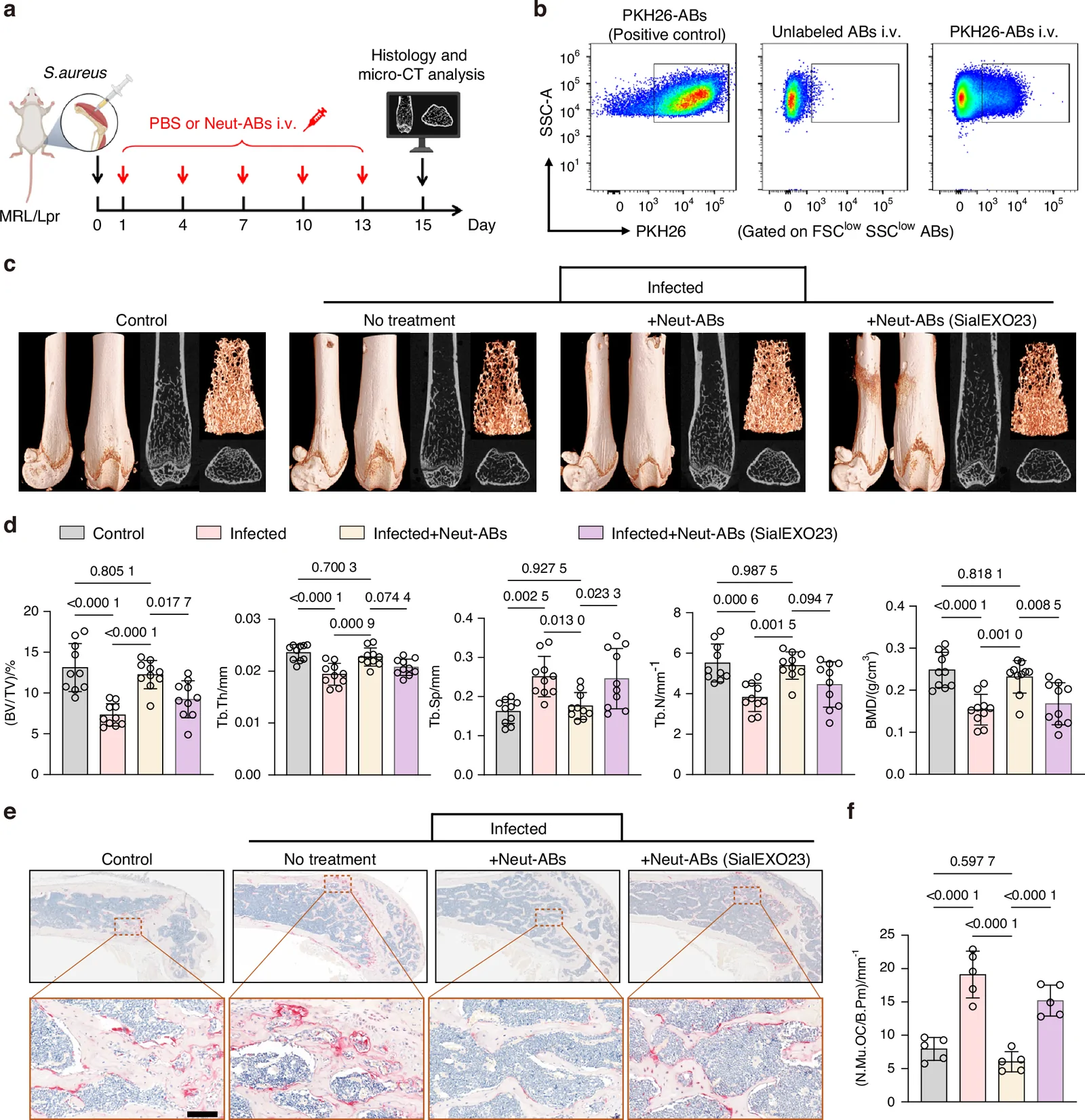
Neutrophil-derived apoptotic bodies protect against bone loss in infectious osteomyelitis. **a** Schematic illustration of the Neut-ABs delivery regimen with intravenous injections administered every 3 days for a total of five doses followed by sacrifice and evaluation. **b** Representative flow cytometry plots of PKH26-labeled Neut-ABs (left panel) and flow cytometric analysis of bone marrow 2 h after intravenous injection of PKH26-labeled or unlabeled Neut-ABs (right two panels). **c**, **d** Representative micro-CT images of femurs from infected mice treated with different treatment groups (**c**). Quantitative micro-CT analysis of bone parameters, including BV/TV, BMD, Tb.Sp, Tb.N and Tb.Th (**d**, *n* = 10). **e**, **f** Representative TRAP-stained histological sections showing osteoclasts in femurs from different treatment groups (**e**, scale bar = 100 µm). Quantification of osteoclast numbers (**f**, *n* = 5), expressed as N.Mu.OC/B.Pm. Data are presented as mean ± SD, with statistical significance (exact *P* values shown in the figure) determined by one-way ANOVA followed by Tukey’s multiple comparisons test (**d** and **f**). See also Fig. S7

To evaluate the therapeutic potential of Neut-ABs in vivo, we examined whether their delivery could attenuate the pronounced bone loss that develops in Lpr mice following *Staphylococcus aureus*–induced osteomyelitis. As illustrated schematically, mice received intravenous injections of either untreated Neut-ABs, SialEXO 2-3-treated Neut-ABs, or PBS every three days starting from day 1 after infection. Each injection contained 5 × 10^7^ Neut-ABs, and bone volume was evaluated by micro-CT and histological analysis two weeks post-infection (Fig. 6a). Flow cytometric analysis confirmed that intravenously administered Neut-ABs were detectable in the bone marrow niche (Fig. 6b).

PBS-treated Lpr mice developed severe trabecular bone loss after infection, demonstrating the destructive impact of osteomyelitis. By contrast, repeated infusion of untreated Neut-ABs preserved bone architecture and conferred robust protection against osteolysis (Fig. 6c). Quantitative micro-CT analysis demonstrated that Neut-ABs treatment significantly improved BV/TV, BMD, Tb.Th, and Tb.N, while reducing Tb.Sp (Fig. 6d). In these parameters, infected Lpr mice treated with Neut-ABs approached levels observed in infected Mpj controls (Fig. 6c, d; Fig. 2g). Importantly, pretreatment of Neut-ABs with neuraminidase markedly diminished their bone-protective effect, underscoring that α2,3-sialylation is essential for their decoy function in blocking osteoclast activation.

Histological analyses provided mechanistic insights into the bone-protective effects of Neut-ABs. Infected Lpr mice exhibited markedly increased numbers of TRAP^+^ osteoclasts (Fig. 6e, f), consistent with enhanced bone resorption and localized bone erosion. Administration of Neut-ABs significantly reduced both osteoclast number and activity, whereas Neut-ABs pretreated with neuraminidase failed to produce this inhibitory effect (Fig. 6e, f). Although Gram staining did not detect overt bacterial aggregates in the bone or bone marrow of Lpr mice (Fig. S7a), CFU quantification indicated that Neut-ABs treatment did not alter bacterial burden or enhance bacterial clearance (Fig. S7b). IHC analysis of the osteoblast marker osteocalcin (OCN) showed no significant differences in osteoblast activity (Fig. S7c, S7d). These observations indicate that Neut-ABs exert a selective inhibitory effect on osteoclast-mediated bone resorption without acting through pathogen clearance or the function of osteoblasts.

## Discussion

Neutrophils are among the most abundant innate immune cells in the bone marrow and are traditionally recognized for their roles in host defense and inflammatory regulation.^54^ While their rapid turnover and apoptosis are well-characterized, the downstream consequences of neutrophil apoptosis for skeletal homeostasis have remained largely unexplored.^55^ Our findings reveal that Neut-ABs represent a previously unappreciated mechanism linking innate immune cell turnover to bone integrity. Infection-driven neutrophil apoptosis generates Neut-ABs enriched in α2,3-sialylated Tlr2, which act as decoy receptors to inhibit Siglec15–Tlr2 signaling in osteoclast precursors. By suppressing precursor fusion and *Nfatc1* activation, Neut-ABs selectively limit osteoclast-mediated bone resorption without apparent effects on osteoblast function or bacterial clearance. These data extend the conceptual framework of immune cell–bone crosstalk, demonstrating that neutrophil apoptosis not only resolves inflammation but also contributes directly to skeletal homeostasis.

Extracellular vesicles are increasingly recognized as active mediators of intercellular communication, influencing immune modulation, tissue repair, and homeostasis across diverse systems.^56^ While macrophage- and tumor-derived apoptotic bodies have been linked to the regulation of inflammation and immune surveillance,^27^ the role of neutrophil-derived vesicles in skeletal physiology has remained speculative.^18^ Our findings suggest that Neut-ABs expand this conceptual framework by revealing how immune cell turnover can directly shape bone remodeling. Sialylation emerges as a central determinant of vesicle function: sialic acids are widely recognized as key mediators of self–non-self discrimination and receptor–ligand interactions in the immune system.^57,58^ Extending this principle to the skeletal niche, we show that α2,3-linked sialylation of Tlr2 endows Neut-ABs with a decoy property, enabling them to compete with endogenous osteoclast signals and thereby constrain osteoclastogenesis. In addition to the Siglec15–Tlr2 axis, other sialylated surface molecules that have been reported to be recognized by Siglec15 and associated with osteoclast differentiation, such as CD44 and LRP1, may also contribute to the inhibitory effects of Neut-ABs.^49,59^ Importantly, Neut-ABs restrain osteoclast activity without affecting osteoblast function or bacterial clearance, revealing a subtle form of regulation within the bone marrow niche. Sialylation, as a post-translational modification, confers functional specificity to these vesicles, enabling them to act as natural decoys that fine-tune receptor signaling in osteoclast precursors.

The decoy mechanism observed in Neut-ABs illustrates a broader principle: apoptotic bodies and extracellular vesicles can transmit functional signals to neighboring cells, shaping tissue remodeling, repair, and homeostasis in environments with rapid immune cell turnover. This perspective, increasingly supported by recent studies,^27,60,61^ frames apoptosis not merely as a terminal event but as an active form of intercellular communication with context-dependent effects. Importantly, this decoy mechanism operates within the RANKL/OPG framework. RANKL–RANK signaling drives osteoclast lineage commitment and differentiation, while sialylation-dependent Siglec15–Tlr2 signaling regulates a later step, namely osteoclast precursor fusion and maturation. In this context, Neut-ABs do not override canonical RANKL-driven differentiation but fine-tune osteoclastogenic output by constraining cell–cell fusion. From a translational standpoint, Neut-ABs highlight the therapeutic potential of vesicle-based interventions. Apoptotic bodies are inherently stable, minimally immunogenic, and scalable, while their activity can be modulated through specific glycosylation patterns.^62^ Engineering vesicles with defined post-translational modifications may allow precise control over osteoclast activity, offering a biologically elegant alternative or complement to conventional antiresorptive therapies. Such strategies could be particularly valuable in conditions where maintaining bone quality and repair capacity is critical, including inflammatory arthritis, osteoporosis, or tumor-induced osteolysis.

Ultimately, these findings expand the conceptual landscape of immune–skeletal crosstalk. Neutrophil apoptosis emerges not only as a mechanism for resolving inflammation but also as a source of instructive signals that preserve skeletal integrity. By demonstrating that innate immune turnover can directly influence tissue homeostasis through vesicle-mediated decoy signaling, our work positions apoptotic bodies as both mechanistic mediators and potential therapeutic agents, opening new avenues for understanding and harnessing immune-driven regulation of organ function.

## Materials and methods

### Mice

All animal procedures were performed in accordance with institutional ethical guidelines and approved by the Institutional Animal Care and Use Committee of Army Medical University. Male mice aged 8–10 weeks were used in all experiments. C57BL/6 J mice were obtained from the Southwest Hospital Animal Center (Chongqing, China), while *Tlr2*^*−/−*^, MRL/MpJ, and MRL/MpJ-Faslpr/J (MRL/Lpr) mice were purchased from Cyagen Biotechnology Co., Ltd. (Suzhou, China). All mice were housed under specific pathogen–free (SPF) conditions with controlled temperature and a 12-h light/dark cycle and had free access to food and water.

### Isolation and culture of bone marrow neutrophils

Bone marrow neutrophils were isolated from mice by negative selection using the EasySep™ Mouse Neutrophil Enrichment Kit (STEMCELL Technologies) according to the manufacturer’s instructions. Purity was assessed by flow cytometry, with neutrophils defined as Ly6G⁺CD11b⁺ cells comprising >90% of the population (see Fig. S1A). Isolated neutrophils were cultured in RPMI-1640 medium supplemented with 10% fetal bovine serum and 1% penicillin/streptomycin for the indicated time points.

### Isolation and culture of BMSCs

Bone marrow-derived mesenchymal stem cells (BMSCs) were isolated from femurs and tibias of mice. Briefly, bone marrow cells were flushed from the hind limbs and centrifuged to remove debris. The cell pellet was treated with red blood cell lysis buffer for 5 min at room temperature to eliminate erythrocytes, followed by centrifugation and resuspension in complete α-MEM. The cell suspension was filtered through a 70 μm cell strainer and plated. Cells were allowed to adhere for 24 h, after which non-adherent cells were removed by discarding the supernatant. Adherent BMSCs were maintained in α-MEM supplemented with 10% fetal bovine serum and 1% penicillin–streptomycin at 37 °C in a humidified 5% CO₂ atmosphere. Medium was changed every 3 days, and cells between passages 2–3 were used for subsequent experiments.

### Wright–Giemsa staining

Suspended cells were collected and dropped onto clean glass slides to prepare thin smears. The smears were air-dried at room temperature. Staining was performed using a Wright–Giemsa Stain Kit (BASO, Zhuhai, China) according to the manufacturer’s instructions. Briefly, solution A was added to cover the smear for 1 min, followed by the addition of 2–3 volumes of solution B, mixing thoroughly for 5 min. After staining, slides were gently rinsed with distilled water, air-dried, and examined under a light microscope. Morphological analysis and imaging were performed to assess cellular features.

### Transmission electron microscopy of cells

Suspended cells were collected and washed twice with cold PBS. Cells were then fixed in 2.5% glutaraldehyde in PBS at 4 °C for 2–4 h, followed by post-fixation in 1% osmium tetroxide for 1–2 h at room temperature. After thorough rinsing with PBS, samples were dehydrated through a graded ethanol series, infiltrated, and embedded in epoxy resin. Ultrathin sections (50–80 nm) were cut using an ultramicrotome, mounted on copper grids, and stained with uranyl acetate and lead citrate. Sections were examined using a transmission electron microscope (FEI TECNAI G2 12, Thermo Fisher Scientific) at an accelerating voltage of 100 kV, and representative images were captured for morphological analysis.

### Isolation of apoptotic bodies

Apoptotic bodies were isolated following standard protocols. Briefly, neutrophils were cultured in vitro for 30 h. Cells were collected and centrifuged at 300 × *g* for 5 min twice to remove intact cells. The resulting supernatant was then centrifuged at 3 500 × *g* for 30 min to collect the pellet as neutrophil-derived apoptotic bodies (see Fig. S1D). To generate differentially treated Neut-ABs, samples were incubated with SialEXO 2–3 (Genovis, 1 U/mL) for 30 min to enzymatically remove α2,3-linked sialic acid modifications, or with excess MAL II lectin (20 μg/mL, Vector Laboratories) for 30 min to block surface α2,3-linked sialic acids. Apoptotic bodies derived from *Tlr2*^*−/−*^ neutrophils were used as TLR2-deficient controls.

For BMSC-derived apoptotic bodies, BMSCs were induced to undergo apoptosis using staurosporine (200 nmol/L, MedChemExpress) for 12 h. The culture medium was then processed using the same centrifugation protocol to collect BMSC-derived apoptotic bodies.

### Immunofluorescence staining

Suspended neutrophils or isolated neutrophil-derived apoptotic bodies were washed with PBS and resuspended in Annexin V binding buffer. Cells or apoptotic bodies were incubated with Annexin V-FITC (BioLegend) and Dil (Sangon Biotech) according to the manufacturer’s instructions. After incubation for 20 min at room temperature in the dark, samples were washed with binding buffer, mounted onto glass slides, and imaged using a confocal microscope.

Osteoclasts were fixed with 4% paraformaldehyde for 10 min at room temperature and blocked with 5% BSA in PBS for 30 min. Cells were incubated overnight at 4 °C with rabbit anti-vinculin antibody (Abcam), followed by incubation for 1 h at room temperature with Goat Anti-Rabbit IgG H&L (Alexa Fluor® 594, Abcam). Subsequently, cells were incubated with Phalloidin-iFluor 488 (Abcam) for 30 min to label the F-actin cytoskeleton, and nuclei were counterstained with DAPI. Images were acquired using a confocal microscope to assess F-actin organization.

### Western blot and lectin blot

Cell lysates were resolved by 10% SDS-PAGE and transferred onto 0.45 μm PVDF membranes. Membranes were blocked with 5% non-fat milk in TBST (TBS with 0.1% Tween-20) for 1 h at room temperature, and then incubated overnight at 4 °C with the following primary antibodies: Caspase-3 (Abcam, 1:1 000), TLR2 (Santa Cruz, 1:200), ST3GAL1 (ABclone, 1:1 000), MyD88 (Proteintech, 1:1 000), DAP12 (Cell Signaling Technology, 1:1 000), IκB-α (MedChemExpress, 1:1 000), NF-κB p65 (Proteintech, 1:1 000), Lamin B1 (Proteintech, 1:1 000), Siglec15 (Kleanab, 1:1 000), Syk (Proteintech, 1:1 000), and Phospho-SYK (Tyr323) (Proteintech, 1:1 000). After three washes with TBST, membranes were incubated with HRP-conjugated secondary antibodies (1:5 000, Affinity Biosciences) for 1 h at room temperature. Protein signals were visualized using enhanced chemiluminescence (ECL, Thermo Fisher) and imaged with a ChemiDoc Imaging System (Bio-Rad). Band intensities were quantified using ImageJ and normalized to β-actin.

For detection of α2,3-linked sialic acids, membranes were incubated with MAL-II lectin (5 μg/mL, Vector Laboratories) followed by HRP-conjugated Streptavidin (Proteintech, 1:5 000) in place of primary and secondary antibodies, respectively.

For immunoprecipitation, target proteins were precipitated using the Universal IP/Co-IP Toolkit (Magnetic Beads) (Abbkine, Wuhan, China) according to the manufacturer’s instructions, followed by Western blot or lectin blot analysis.

Nuclear and cytoplasmic proteins were extracted using the Nuclear and Cytoplasmic Protein Extraction Kit (Beyotime) according to the manufacturer’s instructions. β-actin and Lamin B1 were used as loading controls for cytoplasmic and nuclear proteins, respectively.

Specifically, for the analysis of proteins from apoptotic neutrophils (0–48 h), β-actin was not used as a loading control due to its degradation; equal amounts of protein (30 μg) were loaded per lane for the experiments.

### Flow cytometry analysis

To assess neutrophil purity, bone marrow–derived neutrophils were incubated with anti-Ly6G-PE (BioLegend) and anti-CD11b-FITC (BioLegend) antibodies for 30 min at 4°C in the dark. Samples were analyzed using a BD LSRFortessa™ flow cytometer, and neutrophils were identified as CD11b⁺Ly6G⁺ cells. For apoptosis analysis of cultured neutrophils, cells were resuspended in Annexin V binding buffer and stained with Annexin V-FITC for 15 min at room temperature in the dark, followed by propidium iodide staining for 5 min, and analyzed by flow cytometry. For flow cytometric analysis of apoptotic bodies, samples were stained with Annexin V-FITC and Ly6G to identify neutrophil-derived apoptotic bodies. Culture medium was used as a background control, and 1 μm and 5 μm flow cytometry counting beads (Thermo Fisher) were employed to assist in size-based discrimination and quantification.

### Negative-staining transmission electron microscopy

Suspended apoptotic bodies were collected and washed twice with cold PBS. A small volume of the sample (8–10 µL) was placed onto a formvar/carbon-coated copper grid that had been laid on a sealing film in a culture dish, and allowed to adsorb for 10 min. Excess liquid was gently removed using filter paper. Subsequently, 10 µL of 2% (w/v) uranyl acetate staining solution was added to the grid and incubated for approximately 2 min. Excess stain was removed with filter paper, and the grids were air-dried at room temperature for 10–20 min. Dried grids were examined using a transmission electron microscope (FEI TECNAI G2 12, Thermo Fisher Scientific) at an accelerating voltage of 100 kV, and representative images were captured for morphological analysis.

### Murine model of acute osteomyelitis

Acute osteomyelitis was induced in 8–10-week-old mice under institutional animal ethics approval. The model was based on a previously reported protocol^28^ with minor modifications. Specifically, following inhalational anesthesia with isoflurane, a unicortical bone defect was surgically created in the proximal femur using a 29-gauge needle to generate a minimally invasive microchannel. Subsequently, 2 μL of PBS containing 1 × 10⁶ CFU *Staphylococcus aureus* USA300, or PBS alone (sham control), was injected into the femoral medullary canal through the defect using a 2.5 μL Hamilton microsyringe fitted with a 33-gauge needle. At 24 h post-infection, bone marrow cells were analyzed by flow cytometry. Briefly, hind limbs were harvested, femoral bone marrow cells were flushed, treated with red blood cell lysis buffer, filtered, and stained with Annexin V-FITC and anti-Ly6G-PE in the dark prior to flow cytometric analysis. On day 3 post-infection, colony-forming units (CFUs) in bone marrow were quantified. On day 15 post-infection, bone loss was evaluated using micro-CT followed by histological assessment. Because bacterial injection through the proximal femoral defect inevitably caused cortical bone damage at the injection site, analysis was focused on trabecular bone structures in the distal femur, which were unaffected by surgery. For Neut-ABs treatment, apoptotic bodies were prepared as described above, quantified, and administered via tail vein injection every 3 days (5 × 10⁷ particles per injection).

### Micro-computed tomography analysis

Bone architectural alterations in *Staphylococcus aureus*–infected femurs were assessed using high-resolution micro-computed tomography with a SkyScan 1272 system (Bruker, Kontich, Belgium). Femurs were fixed in 4% paraformaldehyde at 4 °C for 72 h, thoroughly rinsed with PBS, and then subjected to scanning at 80 kV and 165 μA with an isotropic voxel size of 7 μm. A 4-mm region of interest (ROI) encompassing the distal metaphysis was selected for quantitative analysis. Raw projection images were reconstructed into three-dimensional volumes using NRecon software (Bruker), and structural morphometric parameters were computed with CT Analyzer (CTAn, v1.18.8, Bruker). The following indices were quantified: bone mineral density (BMD), bone volume fraction (BV/TV), trabecular number (Tb.N), trabecular thickness (Tb.Th), and trabecular separation (Tb.Sp). Three-dimensional anatomical renderings were generated using CTVol software (Bruker), and osteolytic lesions were visually assessed on reconstructed coronal and sagittal planes. All scanning and analysis procedures adhered to standardized guidelines established by the American Society for Bone and Mineral Research (ASBMR), ensuring methodological consistency and reproducibility.

### TRAP staining and osteoclast quantification

For in vitro osteoclast differentiation assays, cells were fixed with 4% paraformaldehyde for 10 min and stained using a tartrate-resistant acid phosphatase (TRAP) staining kit (GENMED) according to the manufacturer’s instructions. TRAP⁺ multinucleated cells containing ≥3 nuclei were identified as osteoclasts and counted under a light microscope. For histological analysis, decalcified femoral sections were prepared, and TRAP staining was performed using the same kit following the manufacturer’s protocol. TRAP⁺ multinucleated osteoclasts were counted along the trabecular bone surface in the distal femur. Osteoclast number was expressed as the number of TRAP⁺ cells per millimeter of bone surface.

### Immunohistochemistry

Decalcified femoral sections were deparaffinized, rehydrated, and subjected to antigen retrieval with proteinase K for 30 min. Endogenous peroxidase activity was quenched with 3% hydrogen peroxide for 10 min, followed by blocking with 5% goat serum in PBS for 30 min at room temperature. Sections were incubated overnight at 4 °C with rabbit anti-OCN antibody (ABclonal, 1:100), followed by HRP-conjugated goat anti-rabbit IgG secondary antibody for 1 h at room temperature. Staining was developed using DAB substrate and counterstained with hematoxylin. For evaluation, representative images were captured under a light microscope. OCN expression was quantified using ImageJ software by calculating the average optical density (AOD) of positive staining in the trabecular bone region of the distal femur.

### In vitro BMM and osteoclast differentiation

Bone marrow cells were flushed from the femurs and tibias of mice, subjected to red blood cell lysis, and filtered through a 70 µm strainer. Cells were cultured in αMEM supplemented with 10% FBS and 1% penicillin/streptomycin. After 24 h, non-adherent cells in the supernatant were collected and cultured in the presence of M-CSF (25 ng/mL, R&D Systems) to generate bone marrow–derived macrophages (BMMs). For osteoclast differentiation, BMMs were cultured with M-CSF (25 ng/mL, R&D Systems) for 2 days, followed by stimulation with M-CSF (25 ng/mL, R&D Systems) and RANKL (50 ng/mL, R&D Systems) for an additional 4–5 days until multinucleated osteoclasts were formed. For apoptotic bodies intervention experiments, Neut-ABs were isolated and quantified as described above. Neut-ABs were added to osteoclast precursor cultures after 1 day of RANKL stimulation at a ratio of 10:1 (ABs:cells). Control cultures received equal volumes of PBS. Osteoclast formation was assessed by TRAP staining once multinucleated mature osteoclasts appeared in the control group.

### Bone resorption assay and scanning electron microscopy analysis

Bone slices (Bone Slice, Denmark) were placed in 96-well plates. Osteoclast precursors were seeded onto the bone slices and cultured as described above, with parallel wells without bone slices included as controls to monitor osteoclast formation. Culture medium containing M-CSF and RANKL was replaced every 2 days. After osteoclast formation was established, cells were maintained on bone slices for an additional 7 days to allow bone resorption. Bone slices were then removed from the wells and washed thoroughly with PBS to remove residual cells. For surface imaging, bone slices were prepared for scanning electron microscopy by fixation, dehydration through a graded ethanol series, and air-drying. Samples were subsequently mounted on stubs, sputter-coated with gold, and imaged using a scanning electron microscope to assess resorption pits.

### Sample preparation for bulk RNA-seq and proteomic analysis

Fresh neutrophils, apoptotic neutrophils, and neutrophil-derived apoptotic bodies were prepared as described above, with three biological replicates per group. For bulk RNA-seq, cells were lysed in 1 mL TRIzol reagent (Thermo Fisher Scientific) and stored at −80 °C. For proteomic analysis, cell pellets were collected, supernatants removed, and pellets stored at −80 °C. Samples were then shipped to Metware Co., Ltd. (Wuhan, China), which performed RNA and protein extraction and generated raw expression matrices. All downstream data processing, integration, and statistical analyses were conducted using custom scripts in RStudio. The raw sequence data reported in this paper have been deposited in the Genome Sequence Archive in National Genomics Data Center, China National Center for Bioinformation / Beijing Institute of Genomics, Chinese Academy of Sciences (GSA: CRA031784) that are publicly accessible at https://ngdc.cncb.ac.cn/gsa. The mass spectrometry proteomics data have been deposited to the ProteomeXchange Consortium (https://proteomecentral.proteomexchange.org) via the iProX partner repository with the dataset identifier PXD069804.

### Molecular docking

The sequences of the target proteins were obtained from Uniprot and subsequently modeled using AlphaFold3, an advanced deep-learning-based protein structure prediction tool. AlphaFold3 employs an end-to-end transformer-based architecture, leveraging both evolutionary multiple sequence alignments (MSAs) and physical constraints to predict highly accurate three-dimensional protein structures. The model integrates an attention-based deep neural network with structural templates to refine its predictions, enabling the accurate determination of protein folding and domain organization. After conducting structural predictions, we also utilized this to conduct docking studies on the model’s target proteins. The relevant modifications were generated by model reconstruction using PyMol and python. The docking results were analyzed and visualized using PyMOL 2.6.1.

### Quantitative real time PCR

For qRT-PCR analysis, total RNA was isolated using the TRIzol reagent (Invitrogen) according to the manufacturer’s instructions. RNA purity and concentration were assessed with a NanoDrop 2000 spectrophotometer (Thermo Fisher Scientific), ensuring an A260/A280 ratio between 1.8 and 2.0. 1 μg of total RNA was reverse-transcribed into cDNA using the PrimeScript RT Reagent Kit with gDNA Eraser (Takara). qRT-PCR was then performed using TB Green Premix Ex Taq (Takara) on a CFX96 Touch Real-Time PCR Detection System (Bio-Rad) under standard cycling conditions. *Gapdh* served as the internal control. All reactions were run in technical triplicates to ensure reproducibility. Primer sequences for *Nfatc1* and *Gapdh* were listed in the key resources table in the supplementary file.

### Quantification and statistical analysis

Image quantifications were performed using ImageJ (NIH). Statistical analyses were conducted using GraphPad Prism 9.5. Two-tailed unpaired Student’s *t*-tests were used for single comparisons. For multiple group comparisons, one-way or two-way ANOVA with appropriate post hoc tests (as specified in figure legends) were applied. Data are presented as mean ± standard deviation (SD). A *P* value less than 0.05 was considered statistically significant. Details on biological replicates and specific statistical tests are provided in the figure legends.

## Supplementary information

Supplemental figure 1-7
