# Supplementary material for: Neutrophil-derived apoptotic bodies function as decoy signals to preserve bone integrity during infectious osteomyelitis
Source: Bone Res. 2026 Aug 25;14:90. doi: 10.1038/s41413-026-00571-z (PMC13503865; doi:10.1038/s41413-026-00571-z)
Supplement: Supplementary file 1 [file 41413_2026_571_MOESM1_ESM.docx]

**Figure S1. Supplementary illustration of extraction and morphological features of neutrophils and neutrophil-derived apoptotic bodies, related to Figure 1.**

**(a)** Schematic illustration of neutrophil isolation and representative flow cytometric analysis confirming purity of freshly isolated neutrophils.

**(b)** Wright–Giemsa staining of neutrophils from bone marrow (BM) and peripheral blood, showing band cells and segmented nuclei. Morphology of bone marrow neutrophils after 24 h and 48 h of culture is also shown. Scale bar = 5 µm.

**(c)** Representative transmission electron microscopy images showing membrane budding of neutrophils. Scale bar = 5 µm.

**(d)** Schematic illustration of the extraction process for neutrophil-derived apoptotic bodies.

**(e–g)** Representative immunofluorescence images of apoptotic neutrophils and neutrophil-derived apoptotic bodies. Green, Annexin V; red, Dil (e, scale bar = 5 µm). Diameters of apoptotic neutrophils and neutrophil-derived apoptotic bodies measured by confocal microscopy (f, g).

**Figure S2. Osteomyelitis model establishment and inflammatory response in MRL mice, related to Figure 2.**

**(a**) Schematic illustration of the osteomyelitis mouse model. Bacteria were injected into the proximal femur. The region of analysis was the trabecular bone in the distal femur, avoiding cortical bone damage at the injection site.

**(b)** Representative immunofluorescence images of bone marrow sections from steady-state and *S. aureus*–induced osteomyelitis mice, showing Ly6G⁺ neutrophils (red), TUNEL⁺ apoptotic cells (green), and nuclei counterstained with DAPI (blue). Quantification of Ly6G⁺TUNEL⁺ cells in bone marrow sections (n = 8). Scale bar = 50 µm.

**(c–f)** Representative images of male C57BL/6, MRL/Mpj, and MRL/Lpr mice (c), along with body weight measurements (d, n = 9). Representative micro-CT images of femurs (e) and corresponding quantitative analysis of bone parameters (f, n = 5).

**(g)** Flow cytometry gating strategy for bone marrow cells.

Data are presented as mean ± SD. Statistical significance was determined by one-way ANOVA followed by Tukey’s multiple comparisons test (d and f), or by unpaired two-tailed Student’s t-test (b).

**Figure S3. Systemic and skeletal characterization of osteomyelitis in MRL mice, related to Figure 2.**

**(a and b)** Flow cytometric analysis of bone marrow neutrophils. Representative dot plots (a) and quantification of neutrophil percentages (b, n = 8).

**(c)** Quantification of bacterial colony-forming units in bone marrow from Mpj and Lpr mice (n = 5).

**(d)** Quantification of serum TNF-α and IL-1β levels in Mpj and Lpr mice (n = 5).

**(e)** Representative images of renal sections from C57BL/6 mice at baseline and from MRL/Mpj and MRL/Lpr mice at baseline and 14 days post-*S. aureus* infection. Scale bar = 50 µm

**(f and g)** IHC analysis of osteoblast activity in femoral sections. Representative OCN-stained images (f, scale bar = 100 µm) and quantification of staining intensity (g, n = 4).

Data are presented as mean ± SD. Statistical significance was determined by one-way ANOVA followed by Tukey’s multiple comparisons test (b, c, d and g).

**Figure S4. Supplementary analysis of integrated transcriptomic and proteomic data, related to Figure 3.**

**(a)** Principal component analysis of bulk transcriptomic and proteomic datasets.

**(b and c)** Heatmap visualization of key transcriptional gene modules in neutrophils, including anti-apoptotic genes, pro-apoptotic genes, senescence-related genes, and necrosis pathways (b), as well as reported redundant genes (c).

**(d)** Proteomic analysis showing the corresponding proteins detected for the gene modules in panels B and C, as well as neutrophil markers and characteristic proteins such as Mpo, Elane, and Ly6g.

**(e)** Pearson correlation analysis between transcriptomic and proteomic levels to assess consistency between mRNA and protein expression.

**(f)** Up- and down-regulation status of differentially expressed proteins among the three sample groups.

**(g)** Subcellular localization of the top 100 proteins detected in the proteomic dataset.

**(h and i)** Volcano plot (h) and heatmap (i) visualization of differentially expressed proteins among the three sample groups, with proteins of interest highlighted.

**Figure S5. Supplementary illustration of pre-osteoclast recognition and fusion via Siglec15–TLR2, related to Figure 4.**

**(a**) Representative images of osteoclasts during culture with or without sialidase (SialEXO23) treatment. Scale bar = 100 µm.

**(b)** Identification of candidate Siglec15 ligands by proximity labeling, visualized using publicly available data from the ProteomeXchange Consortium (PXD006359).

**(c)** Schematic illustrating the mechanism of pre-osteoclast recognition via Siglec15–TLR2. Adapted from ***Bone Research***, 2022, “Sialylation of TLR2 initiates osteoclast fusion” (doi: 10.1038/s41413-022-00186-0).

**(d)** Heatmap showing mRNA expression levels of *Tlr2*, *Siglec15*, and sialyltransferase family members in fresh neutrophils, apoptotic neutrophils, and neutrophil-derived apoptotic bodies.

**Figure S6. Supplementary illustration of the mechanism by which Neut-ABs block osteoclast recognition and fusion, related to Figure 5.**

**(a–c)** Representative western blot images of Syk and phospho-Syk (a) with corresponding quantification (b, c, n = 3). β-actin and total protein were used as loading controls.

**(d)** Schematic illustration of osteoclast differentiation under conditions of Neut-ABs absence or decoy blockade.

Data are presented as mean ± SD, with statistical significance (exact *p* values shown in the figure) determined by one-way ANOVA followed by Tukey’s multiple comparisons test (b and c).

**Figure S7. Supplementary evaluation of bacterial burden and osteoblast activity in mice treated with Neut-ABs, related to Figure 6.**

**(a)** Representative Gram staining of femoral bone and bone marrow sections. Scale bar = 200 µm.

**(b)** Quantification of bacterial colony-forming units in bone marrow from different treatment groups (n = 5).

**(c and d)** Immunohistochemical staining for OCN in femoral sections (c, scale bar = 100 µm) and quantification of staining intensity (d, n = 5).

Data are presented as mean ± SD, with statistical significance (exact *p* values shown in the figure) determined by one-way ANOVA followed by Tukey’s multiple comparisons test (b and d).

**Key resources table**

| **REAGENT or RESOURCE** | **SOURCE** | **IDENTIFIER** |
| --- | --- | --- |
| **Antibodies** | | |
| FITC Annexin V | Biolegend | Cat# 640905  RRID: AB_2561291 |
| anti-mouse Ly6G-PE | Biolegend | Cat# 127607  RRID: AB_1186104 |
| anti-mouse CD11b-FITC | Biolegend | Cat# 101206  RRID: AB_312789 |
| Anti-Vinculin antibody | Abcam | Cat# ab129002  RRID: AB_11144129 |
| Goat Anti-Rabbit IgG H&L (Alexa Fluor® 594) | Abcam | Cat# ab150080  RRID: AB_2650602 |
| Anti-Caspase-3 antibody | Abcam | Cat# ab32351  RRID: AB_725946 |
| TLR2 (TL2.1) | Santa Cruz Biotechnology | Cat# sc-21759, RRID: AB_628363 |
| ST3GAL1 Polyclonal Antibody | ABclonal | Cat# A11616  RRID: AB_2758638 |
| MYD88 Monoclonal antibody | Proteintech | Cat# 67969-1-Ig RRID: AB_2918720 |
| Phospho-SYK (Tyr323) Recombinant monoclonal antibody | Proteintech | Cat# 83331-1-RR  RRID: AB_3670995 |
| SYK Polyclonal antibody | Proteintech | Cat# 14858-1-AP RRID: AB_2878086 |
| Lamin B1 Polyclonal antibody | Proteintech | Cat# 12987-1-AP  RRID: AB_2136290 |
| NF-κB p65 Recombinant monoclonal antibody | Proteintech | Cat# 80979-1-RR  RRID: AB_2918923 |
| IKB alpha Antibody (YA350) | MedChemExpress | Cat# HY-P80718  RRID: AB_3694198 |
| DAP12 (D7G1X) Rabbit mAb | Cell Signaling Technology | Cat# 12492  RRID: AB_2721120 |
| SIGLEC15 rabbit polyclonal antibody | KleanAB | Cat# P103014  RRID: AB_2510553 |
| Recombinant Anti-beta Actin (HRP Conjugated) | Servicebio | Cat# ZB15001-HRP  RRID: AB_3714580 |
| Osteocalcin (BGLAP) Rabbit mAb | ABclonal | Cat# A20800  RRID: AB_3097802 |
| Goat Anti-Rabbit IgG (H+L) HRP | Affinity Biosciences | Cat# S0001  RRID: AB_2839429 |
| **Bacterial and virus strains** | | |
| *Staphylococcus aureus* USA300 | ATCC | ATCC 43300 |
| **Chemicals, peptides, and recombinant proteins** | | |
| Propidium Iodide | Biolegend | Cat# 421301 |
| Annexin V Binding Buffer | Biolegend | Cat# 422201 |
| RPMI-1640 medium | Gibco | Cat# 11835030 |
| Bovine Serum Albumin | Sigma-Aldrich | Cat# A2153 |
| Wright Giemsa Stain | BASO | Cat# BA4010 |
| SialEXO 2-3 Lyophilized | Genovis | Cat# G1-SD2-005 |
| Maackia Amurensis Lectin II, Biotinylated | Vector Laboratories | Cat# B-1265-1 |
| HRP-conjugated Streptavidin | Proteintech | Cat# SA00001-0 |
| Staurosporine | MedChemExpress | Cat# HY-15141 |
| DiI (Cell Membrane Orange Fluorescent Probe) | Sangon Biotech | Cat# E607331 |
| DAPI Staining Solution | beyotime | Cat# C1005 |
| Phalloidin iFluor 488 | Abcam | Cat# ab176753 |
| Recombinant Mouse M-CSF Protein | R&D Systems | Cat# 416-ML |
| Recombinant Mouse TRANCE/RANK L/TNFSF11 | R&D Systems | Cat# 462-TEC |
| 4% Paraformaldehyde Fix Solution | Beyotime | Cat# P0099 |
| SDS-PAGE Sample Loading Buffer，5X | Beyotime | Cat# P0015L |
| RIPA Lysis Buffer | Beyotime | Cat# P0013B |
| Phenylmethanesulfonyl fluoride | Beyotime | Cat# ST506 |
| Protease and phosphatase inhibitor cocktail | Beyotime | Cat# P1045 |
| FuturePAGETM 10% | ACE | Cat# ET15010LGel |
| MOPS-SDS Running Buffer | ACE | Cat# BR0001-02 |
| Western Transfer Buffer | Servicebio | Cat# G2148-1L |
| Tween 20 | Beyotime | Cat# ST1726 |
| PBS | Servicebio | Cat# G4202 |
| TB Green® Premix Ex Taq™ II (Tli RNaseH Plus) | TaKaRa | Cat# RR820A |
| PrimeScript™ RT reagent Kit with gDNA Eraser | TaKaRa | Cat# RR047A |
| DEPC-treated Water (DNase、RNase free) | Beyotime | Cat# R0022 |
| TRIzol^TM^ | Invitrogen | Cat# 15596018CN |
| **Critical commercial assays** | | |
| EasySep™ Mouse Neutrophil Enrichment Kit | STEMCELL | Cat# 19762 |
| Red Blood Cell Lysis Buffer | Solarbio | Cat# R1010 |
| Universal IP/Co-IP Toolkit (Magnetic Beads) | Abbkine | Cat# KTD104-CN |
| Nuclear and Cytoplasmic Protein Extraction Kit | Beyotime | Cat# P0028 |
| Flow Cytometry Sub-Micron Size Reference Kit | Thermo Scientific | Cat# F13839 |
| Tartrate Resistant Acid Phosphatase Assay Kit | GENMED | Cat# MS80077.1 |
| Cortical bovine bone slices 0.2mm/0.4mm | Boneslices | Cat# 200/400 |
| Cell Plasma Membrane Staining Kit with PKH26 | Beyotime | Cat# C2071S |
| TUNEL Apoptosis Assay Kit | Beyotime | Cat# C1088 |
| Mouse IL-1 beta ELISA Kit | ABclonal | Cat# RK00006 |
| Mouse TNF-alpha ELISA Kit | ABclonal | Cat# RK00027 |
| **Deposited data** | | |
| Bulk RNA-seq data of neutrophils, apoptotic neutrophils, and neutrophil-derived apoptotic bodies | This study | GSA: CRA031784 |
| Proteomic data of neutrophils, apoptotic neutrophils, and neutrophil-derived apoptotic bodies | This study | PXD069804 |
| **Experimental models: Organisms/strains** | | |
| C57BL/6J mice | Southwest Hospital Animal Center | N/A |
| Tlr2-KO mice | Cyagen | Cat# C001263 |
| MRL/MpJ | Cyagen | Cat# C001245 |
| MRL/Lpr | Cyagen | Cat# C001194 |
| **Oligonucleotides** | | |
| Primers for *Nfatc1*  GGAGAGTCCGAGAATCGAGAT (F)  TTGCAGCTAGGAAGTACGTCT (R) | This paper | N/A |
| Primers for *Gapdh*  TGTGTCCGTCGTGGATCTGA (F)  TTGCTGTTGAAGTCGCAGGAG (R) | This paper | N/A |
| **Software and algorithms** | | |
| ImageJ v2.14.0 | National Institutes of Health | N/A |
| Prism v9.5 | GraphPad | N/A |
| IBM SPSS Statistics v26 | SPSS | N/A |
| ZEISS ZEN lite | Carl Zeiss AG | N/A |
| R v4.4.2 | R Development Core Team | N/A |
| RStudio | Posit | N/A |
| NovoExpress | Agilent | N/A |
| **Other** | | |
| LSM 880 laser scanning confocal microscope | Carl Zeiss | N/A |
| FEI TECNAI G2 12 | Thermo Scientific | N/A |
| BD LSRFortessa^TM^ Cell Analyzer | BD Biosciences | N/A |
| Micro-CT Skyscan 1272 system | Bruker | N/A |
| CT-Analyser (CTAn) v1.18.8 | Bruker | N/A |
